# Reassessing and Updating Regulatory Standards on the Declaration of Storage Conditions in the Product Information of Medicinal Products: An Evidence-Based Proposal under EMA Guidance

**DOI:** 10.1007/s11095-026-04122-3

**Published:** 2026-05-20

**Authors:** Helena Pericão, Henrique Cavaco, Miguel Dias, Luís Sarmento, Mário Magalhães, Bernardo Filipe, Herivelto Vieira, João Rita, Pedro Gonçalves, Matilde Vaz, Beatriz Coimbra, Beatriz Monteiro, Beatriz Gomes, Cátia Josué, Ecaterina Fuior, Lara Penso, Maria Ferreira, Maria Leonor Forjaz, Rodrigo Neves, Sofia Azevedo, Constança Lopes, Sofia Rosário, Rita Leal, Pia Erjavec, Felipe Lima, Rui Loureiro, Gracinda M. M. Sanches-Fernandes

**Affiliations:** 1 iBB, Institute for Bioengineering and Biosciences, Department of Bioengineering, Instituto Superior Técnico, Universidade de Lisboa, Avenida Rovisco Pais, 1049-001 Lisbon, Portugal; 2 Associate Laboratory i4HB—Institute for Health and Bioeconomy at Instituto Superior Técnico, Universidade de Lisboa, Av. Rovisco Pais, 1049-001 Lisbon, Portugal; 3 Faculdade de Farmácia, Universidade de Lisboa, Avenida Professor Gama Pinto, 1649-003 Lisbon, Portugal

**Keywords:** EMA guideline, Labelling, Medicinal products, Stability, Storage conditions

## Abstract

**Purpose:**

The European Medicines Agency (EMA) guideline *Declaration of Storage Conditions in the Product Information of Medicinal Products* (CPMP/QWP/609/96/Rev.2) was last revised in 2007. Since then, advances in stability science, increased climatic variability, and evolving regulatory expectations have highlighted limitations in the framework, particularly the use of vague descriptors such as “room temperature” or “no special storage conditions.” This work reassesses the scientific and regulatory basis for storage statements, integrating current understanding of mean kinetic temperature (MKT), climate-related risks, and developments in ICH and WHO guidance, and proposes a harmonized revision (Rev.3).

**Methods:**

A structured analysis of regulatory and scientific principles was conducted, incorporating MKT, climate-related risks, and developments in ICH and WHO guidance. These elements informed a revised framework and model storage statements, including a decision table linking stability outcomes to label statements.

**Results:**

The proposed update introduces explicit temperature-based statements, strengthens the linkage between stability data and label wording, clarifies requirements for moisture- and light-sensitive products, expands guidance for sterile and reconstituted preparations, and provides contingency instructions for temperature excursions. A decision table mapping stability outcomes to label statements is included to support regulatory implementation.

**Conclusion:**

This revision provides an updated synthesis of EMA, ICH, and WHO requirements, translating regulatory principles into clear storage statements and contingency guidance. It offers a more precise framework reducing ambiguity, supports justification of storage conditions through stability data and MKT-informed risk assessment, aiming to improve understanding among patients and healthcare professionals, strengthening real-world product quality and contributing to future EMA guideline development.

**Highlights:**

- Identifies scientific and regulatory limitations in current “no special storage conditions” terminology;
- Links ICH stability guidelines and climatic variability to precise storage statements;
- Proposes harmonised core label phrases for room temperature, fridge, and freezer;
- Introduces contingency labelling for climate-related and cold-chain excursions.

## Introduction

Quality, safety, and efficacy are the three crucial pillars of medicines [1]. To ensure that they are preserved throughout the product’s shelf life, appropriate storage needs to be guaranteed, by being scientifically justified and clearly communicated [2]. Within the European Union (EU), Directive 2001/83/EC requires that storage conditions aligned with the Summary of Product Characteristics (SmPC) be declared in the package leaflet and, where appropriate, on the package labelling [3]. The EMA guideline CPMP/QWP/609/96/Rev 2 provides the regulatory framework for such declarations [4].

The current version of this guideline, revised in November 2007, is based largely on ICH stability conditions for Climatic Zones I and II, with long-term storage typically at 25°C/60% relative humidity (RH), supported by accelerated and, where applicable, intermediate conditions [4]. These conditions are intended to reflect the mean kinetic temperature (MKT) across Europe.

MKT is a significant single virtual temperature that outlines the cumulative thermal stress of a fluctuating temperature profile using Arrhenius-based kinetics [5]. The MKT is therefore more informative than a simple arithmetic mean when excursions occur, due to weighting higher temperatures disproportionately, consistent with the Arrhenius relationship, and making it more representative of real-world thermal stress [6]. The magnitude of the excursion is impacted by several parameters, besides peak temperature, including its duration, the baseline storage condition, shelf life, and product specification activation energy [7].

Climate change adds another layer of significant relevance. Across the world, storage and transport conditions are becoming more variable, with recurrent heat waves, prolonged hot periods, droughts, and power outages all contributing to the greater likelihood of exposure outside intended storage ranges [8]. In Malawi, the MKT of storage for 27 health facilities was monitored for 9 months and maintained through the usage of thermometers and, to a lesser extent, air conditioners due to electrical problems. Overall, an MKT of 25.3ºC was registered, and 46.15% of the personnel had good knowledge of proper storage conditions [9]. In 154 US households, where medical storage conditions were assessed, only 23.3% (28) had all medications stored appropriately, compromising the stability and sterility of drug products in most households [10]. In China, a cohort of 625 households paid great attention to the expiration date of medicines (37.4%), followed by humidity (7.8%) and storage temperature (7.8%), particularly in the context of refrigerated medication (19.2%) [11].

This review highlights a clear mismatch between the recommended storage conditions instructions and what happens in practice, due to both environmental factors and user behaviour. Even when some level of awareness or control exists, medicinal products are still frequently exposed to conditions outside of their intended range. This supports the idea that current medicinal storage instructions may not be as clear or as practical as they should. Therefore, the literature supports the need for more realistic, harmonized and user-oriented storage statements guidance, giving particular attention to increasing climate variability and real-world handling conditions.

Another challenge is the growing global distribution of time and temperature-sensitive medicinal products, which demands robust cold-chain management. Ensuring product integrity and quality throughout this complex supply chain is often difficult [12]. Guideline CPMP/QWP/609/96/Rev 2 presents some ambiguities and several developments that have occurred since 2007, which justify revisiting this framework. Furthermore, scientific and regulatory advances, including the evolution of ICH stability guidelines (Q1A, Q1B, Q1E), WHO guidance on temperature-sensitive products related to storage and transport, and the review of EU labelling/QRD recommendation, reinforce the necessity of this update. Finally, ambiguous statements, such as “*This medicinal product does not require any special storage conditions*”, should be replaced as they can lead to a subjective individual assessment and misinterpretation of the appropriate storage conditions, which can compromise consistent product quality.

In response to increasing recognition within the scientific and regulatory community, we identified ambiguity in how “normal” storage conditions are perceived and applied, especially regarding products labelled as not requiring special storage conditions. This work reassesses the scientific and regulatory foundations of storage declarations and proposes a structured, harmonised revision of the EMA guideline (Rev.3), focusing on finished medicinal products and patient‑facing communication.

## Methods

### Study Design and Data Sources

**Fig. 1:**
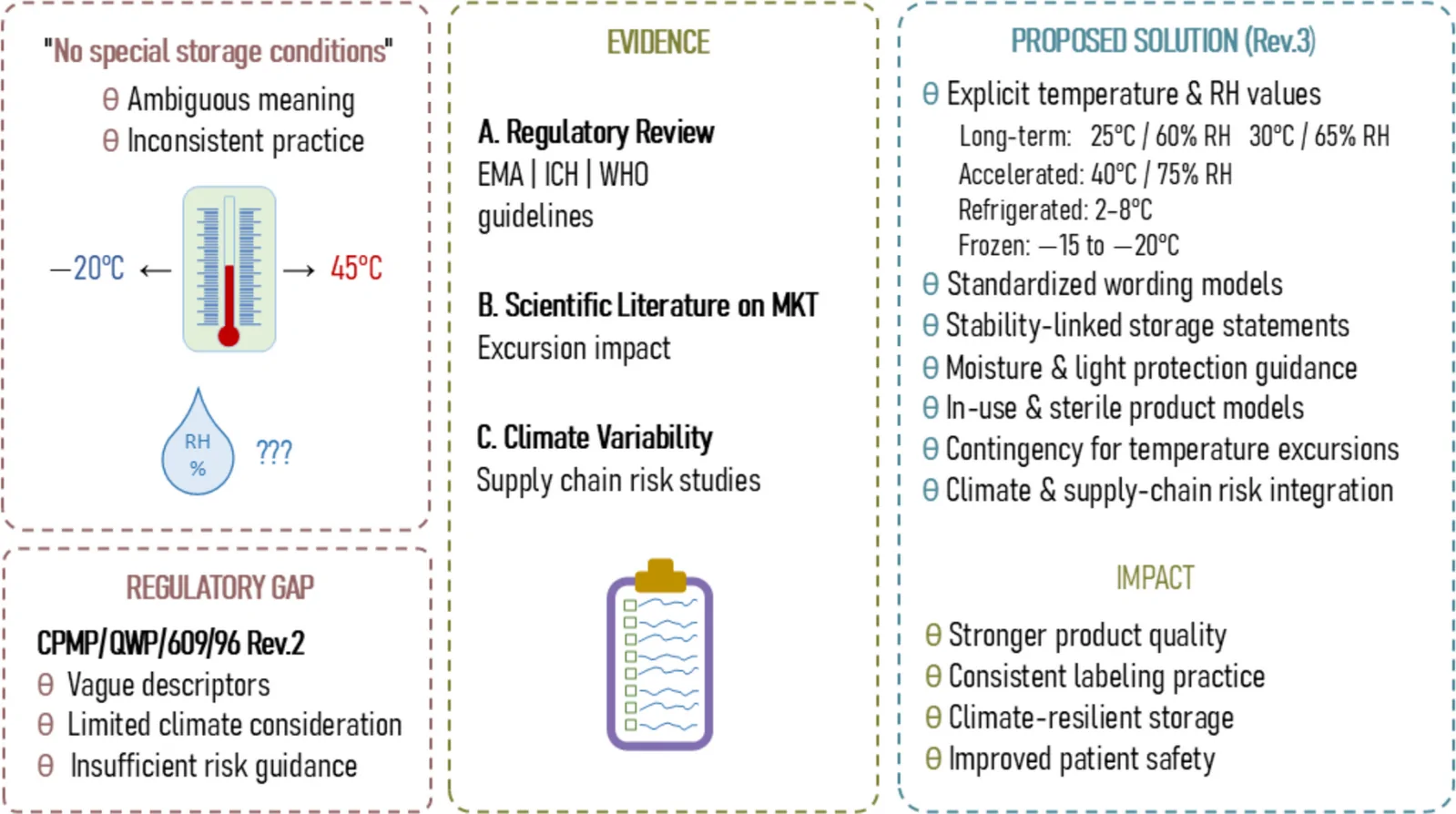
Methodological workflow combining documentary review of EMA, ICH, and WHO guidelines, including development of proposed storage condition statements.

The methodological workflow (Fig. 1) comprised a targeted guideline review of key EMA, ICH, and WHO documents relevant to stability, storage, in-use periods, and labelling guidelines. Evidence from the regulatory and scientific literature review was integrated to develop a draft proposal to revise CPMP/QWP/609/96/Rev.2 to Rev.3, including detailed model storage statements.

### Regulatory Framework Review

A targeted regulatory framework review was performed, focusing on documents relevant to storage conditions, stability, and labelling, from the European Union (EU), International Council for Harmonisation of Technical Requirements for Pharmaceuticals for Human Use (ICH), and World Health Organization (WHO), including the following guidelines present in Table I:

**Table I:** Regulatory Framework Used in the Revision Proposed and their Year of Publishing

EMA: *Human Medicines Evaluation – Recommendations for the Implementation of Exemptions to the Labelling and Package Leaflet Obligations in the Centralised Procedure (QRD Group)* [13]*;*	2019
EMA: *Guideline on Summary of Product Characteristics (SmPC)* [14]*;*	2009
ICH Q1A (R2): *Stability Testing of New Drug Substances and Products* [15]*;*	2003
ICH Q1B: *Photostability Testing of New Active Substances and Medicinal Products* [16]*;*	1998
ICH Q1E: *Evaluation of Stability Data* [17]*;*	2003
ICH E12: *Principles for Clinical Evaluation of New Antihypertensive Drugs* (for contextual regulatory alignment); [18]	2000
EMA: *Guideline on In-use Stability Testing of Human Medicinal Products* [19]*;*	2001
EMA: *Guideline on Maximum Shelf-life for Sterile Products for Human Use after First Opening or Following Reconstitution* [20]*;*	1998
EMA: *Guideline on Stability Testing of Existing Active Ingredients and Related Finished Products* [21]*;*	2023
WHO Technical Report Series No. 961, Annex 9: *Model Guidance for the Storage and Transport of Time- and Temperature-Sensitive Pharmaceutical Products* [22]*;*	2011
WHO Technical Report Series No. 1010, Annex 10: *Stability testing of active pharmaceutical ingredients and finished pharmaceutical products* [23]	2018

These documents were analysed to identify requirements and best practices relevant to defining storage conditions, evaluating stability data, managing in-use periods, and handling temperature excursions, with the intention of providing detailed changes to the EMA guideline CPMP/QWP/609/96/Rev 3. The analysis focused on identifying gaps, inconsistencies, and opportunities for harmonisation.

### Development of Proposed Guideline Revision (CPMP/QWP/609/96/Rev 3)

Based on the documentary review findings, a proposal was drafted to revise the current guideline. The process focused on providing a stronger linkage between the storage statements and stability data, providing explicit temperature and relative humidity (RH) values, providing a clear and harmonised wording for the proposed core storage conditions, providing more detailed information for sensitive product categories (such as sterile products and moisture and light-sensitive products), contingency guidance for possible temperature excursions, providing further alignment with the ICH Q1A definitions of what a “significant change” is, and including a decision table mapping stability outcomes to storage statements.

## Results

### Scientific Basis for Revising Storage Statements

MKT provides a scientifically robust method for integrating temperature fluctuations into stability considerations. Recent studies demonstrate:

- MKT increases significantly with short‑term heat excursions, even when average temperatures remain within acceptable limits;
- Climate‑related events (heatwaves, power outages) are increasingly common and can elevate MKT beyond the stability envelope of many products;
- Real‑world storage conditions in households and healthcare facilities often exceed labelled limits, particularly for products stored at “room temperature”.

These findings reinforce the need for explicit, numerically defined storage statements. Storage deviations can lead to risks such as:

- Accelerated chemical degradation;
- Loss of potency;
- Formation of degradation products;
- Physical instability (e.g., precipitation, phase separation);
- Microbiological risk in sterile or reconstituted products;
- Packaging failures, particularly in semi‑permeable containers.

Since these risks can compromise the efficacy, safety, and quality of medicines, ambiguous statements such as “no special storage conditions” that do not adequately communicate them appropriately must be avoided.

### Proposed Guideline Revision (CPMP/QWP/609/96/Rev 3)

**Fig. 2:**
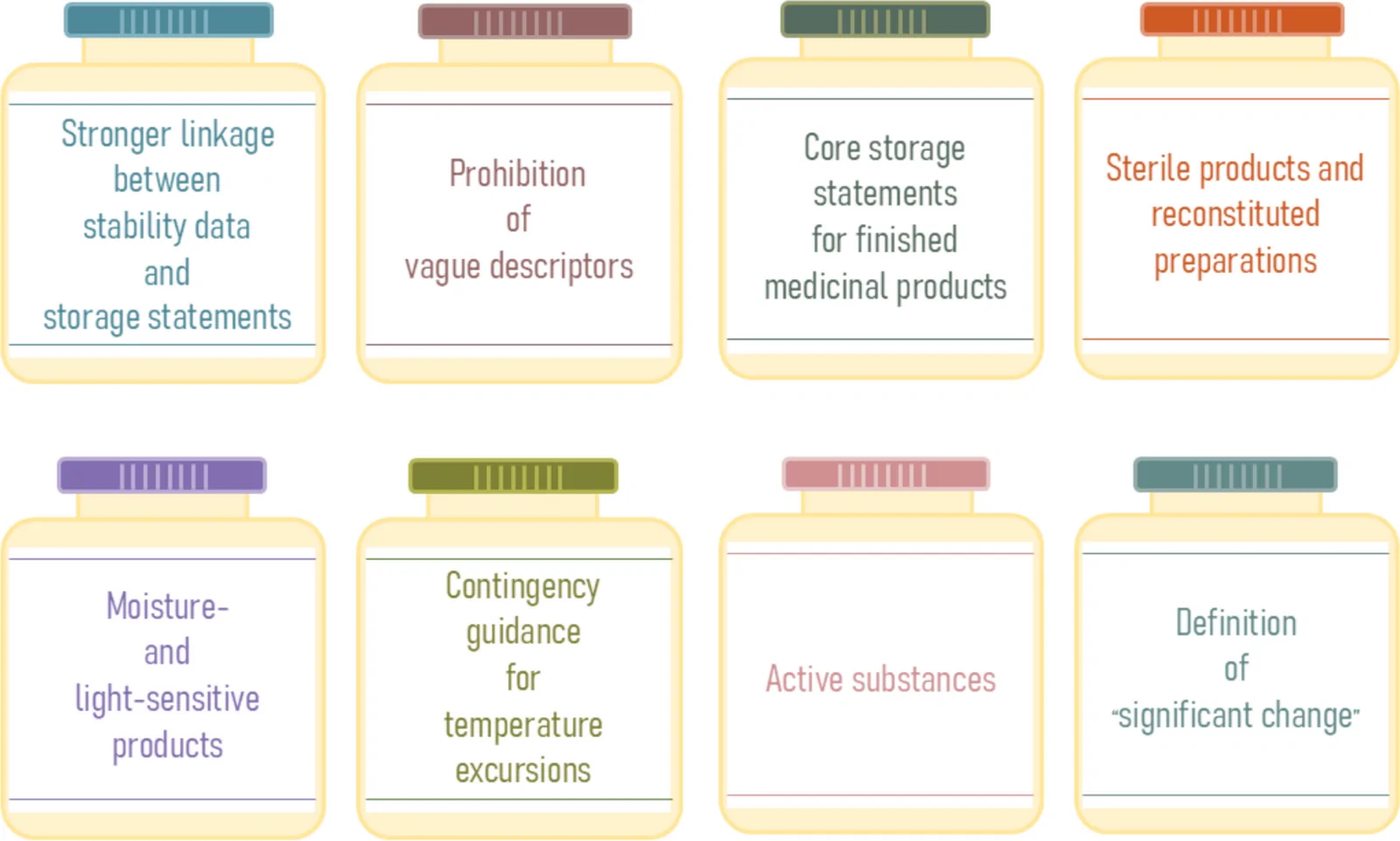
Key elements of the proposed guideline revision (CPMP/QWP/609/96/Rev 3)

The proposed revision restructures and expands the existing guideline (Fig. 2) as follows.

#### Stronger Linkage Between Stability Data and Storage Statements

The proposal emphasises that storage conditions for medicinal products must be scientifically justified and directly supported by ICH-compliant stability studies, including long-term stability studies, typically conducted at 25°C/60% RH (Climatic Zones I/II) or at 30°C/65% RH, when justified. It further requires the use of appropriate intermediate and accelerated conditions and the consideration of contemporary climatic information for the intended distribution geography, including climate variability and climate change.

Applicants are required to justify proposed storage statements using such data and, when applicable, packaging performance data and transport qualification studies. The risk assessment must always be product specific. Lastly, storage statements must not be used to compensate for insufficient stability data (e.g., lack of accelerated or intermediate studies).

#### Prohibition of Vague Descriptors

The use of non-specific terms such as “room temperature” and “ambient conditions” is explicitly deemed unacceptable, as they do not convey precise or measurable parameters for ensuring product quality throughout its shelf life. Therefore, the storage statements must include a limit temperature, such as “Do not store above 25°C” or “Do not store above 30°C” or provide a clearly defined temperature range, like “Store in a refrigerator (2°C to 8°C)”.

Furthermore, where supplementary warnings (e.g., protection from light or moisture) are required, the statement “This medicinal product does not require any special storage conditions” should be revised to “This medicinal product does not require any special temperature storage conditions”.

#### Core Storage Statements for Finished Medicinal Products

The proposal defines standardised storage statements based on demonstrated stability conditions. To support consistent application of these principles, Table II provides a decision matrix mapping typical ICH stability study outcomes to the corresponding proposed storage statements.

**Table II:** Decision Matrix Linking Stability Study Outcomes to Proposed Storage Statements

Stability Data/Study Outcome	Identified Risk	Condition/Trigger	Proposed Storage Statement
Stable at 25°C/60% RH (no significant change)	Minimal Degradation	Standard long-term conditions	Do not store above 25°C
Stable at 30°C/65% RH	Suitable for warm climates	Zone IV conditions	Do not store above 30°C
Degradation observed above 25°C	Heat sensitivity	Increase in impurities > specification	Do not store above 25°C Avoid temperature excursions
Significant degradation with short excursions (e.g. 40°C)	Excursion risk	Repeated temperature spikes	Do not store above 25°C. Excursions above this temperature should be minimized
Hygroscopic API/product	Moisture sensitivity	High relative humidity exposure	Store in a dry place Protect from moisture
Light-sensitive product	Photodegradation	Exposure to light	Store in original package to protect from light
Requires refrigeration (stable at 2–8°C)	Thermal instability at room temp	Degradation above 8°C	Store in a refrigerator (2–8°C). Do not freeze
Freezing causes degradation	Freeze sensitivity	Ice crystal formation	Do not freeze

##### Controlled Room Temperature

Products confirmed stable under long-term conditions of 25°C/60% RH or 30°C/65% RH, together with accelerated studies at 40°C/75% RH, should be labelled “Do not store above 25°C” or “Do not store above 30°C,” as appropriate. When scientifically justified, statements such as “Do not refrigerate or freeze” may also be included when low temperature exposure may cause physical instability.

##### Intermediate Conditions

Products whose stability supports conditions such as 25°C/60% RH (long-term), 30°C/60–65% RH (intermediate), or 30°C/65% RH (long-term), should be labelled “Do not store above 30°C” or “Store below 30°C”. When referring to conditions on refrigeration, similar restrictions should be added, for instance, “Do not refrigerate or freeze”.

##### Refrigerated Products

To ensure long-term stability at 5°C ± 3°C, the product must be stored and transported refrigerated (with the SmPC/PL specifying the range 2°C to 8°C), with the clear instruction “Do not freeze.”

##### Frozen Products

When supported by long-term stability data below 0°C, consistent with ICH freezer conditions, it should specify “Store in a freezer” or “Store and transport frozen” and, when appropriate, specify the range “ − 15°C to − 20°C”.

Alternative phrasing (e.g., “Store below 25°C” *vs.* “Do not store above 25°C”) may be allowed to accommodate linguistic and cultural differences, subject to competent authority approval and consistent use.

#### Sterile Products and Reconstituted Preparations

The revision introduces explicit in-use stability statements. For unpreserved sterile products, aqueously preserved sterile products, and non-aqueous sterile preparations, model statements are provided. On secondary packaging, manufacturers should indicate that, when stored at specific conditions the stability of the product is granted for a defined number of hours or days at a given temperature, including the following statement “If stored under *Z* conditions, stability is granted for *X* hours/days at *Y *°C.”, and in the package leaflet should be included “If stored under *Z* conditions, stability is granted for *X* hours/days at *Y *°C. The user is responsible for maintaining these conditions, otherwise, stability is not guaranteed.”. The variables *X*, *Y,* and *Z* must be defined by the manufacturer based on in-use stability studies.

Additionally, the product must include recommendations advising users to “Open only immediately before use to minimise microbial contamination.”, for products requiring multiple concentrations (e.g. paediatric dosing), the labelling should include “Stability tests were performed and considered valid for the range *X–Y *°C, for *Z* time.”, and for reconstituted products should include “The product, once reconstituted, should have the following appearance: [description]. It must not be stored for more than *Y* [hours/days].”

For unpreserved preparations for infusion or injection, the guideline also recommends their immediate use. When this is not possible, predefined in-use periods, supported by microbiological and stability data, must be stated in statements such as “use within 24h at 2°C to 8°C” may be employed.

#### Moisture and Light-Sensitive Products

The guideline clarifies that storage statements should not compensate for inadequate packaging and that the products should be packaged in containers that ensure stability. Nevertheless, additional labelling may be necessary.

##### Moisture-Sensitive Products

In the case of moisture-sensitive products, impermeable containers generally do not require extra labelling, whereas semi-permeable containers may require additional statements like “Keep the [container] tightly closed” or “Store in the original package.”

Where time-limited stability exists under specific conditions, statements analogous to those for in-use stability may be used.

##### Light-Sensitive Products

Specific model statements are provided by dosage form:

- Tablets: “Protect from light. Keep in the sealed packaging until use.”;
- Creams/gels: “After opening, keep in a place protected from light and use within X days.”;
- Multidose liquids: “Keep in the original packaging and protect from light between uses. After opening, use within X days.”;
- Powders for reconstitution: “After reconstitution, use immediately or keep protected from light for up to X hours.”.

Values for *X*, *Y,* and *Z* must be defined based on stability and photostability data (ICH Q1B).

#### Contingency Guidance for Temperature Excursions

Recognising the increasing risk of climate-related events and cold-chain disruptions, the proposal introduces explicit contingency guidance:

##### General Products

Example leaflet text: “If the product has been exposed to temperatures above 30°C for more than 24h, contact your pharmacist before use.”

The time/temperature thresholds must be product-specific and based on stability data.

##### Refrigerated Products (2–8°C)

The package leaflet should specify alternative temporary storage conditions and safe exposure durations, for example:

“If the product cannot be stored under the recommended conditions, it may be stored under *Z* conditions, for which stability is granted for *X* hours/days at *Y*°C.”

##### Frozen Products (− 15°C to − 20°C)

Guidance for healthcare professionals on excursions may include:

“Store the product in a freezer between A and B °C. If this is not possible, the product may be kept at refrigerator temperature (2°C to 8°C) for up to X hours/days. Any product not used within Y hours/days after removal from the A to B °C range should be discarded.”

Again, *A*, *B*, *X,* and *Y* are derived from dedicated stability studies.

#### Active Substances

Even though the primary focus of the revision is finished medicinal products, the same scientific principles are applied to active substances, but the user context and risk profile differ. Storage declarations for active substances should be based on ICH-compliant stability studies, clearly indicating when refrigerated or frozen storage is required, including explicit temperature ranges, and taking into account the packaging and transport conditions used in routine handling.

#### Definition of “significant change”

The proposal adopts the ICH Q1A (R2) definition of a “significant change” during accelerated stability testing. If a significant change is observed within 6 months under accelerated conditions, intermediate-condition testing becomes mandatory, and at least 6 months of intermediate data must be available at the time of marketing authorisation. Significant change includes a ≥ 5% change in assay or failure to meet potency specifications, any degradation product exceeding its specification, failure in appearance, physical attributes, or functionality, pH outside the specified range, and failure to meet dissolution criteria for 12 dosage units. Such changes may restrict allowable storage conditions and shelf-life, thereby directly influencing the storage statements in product information.

## Discussion

This work highlights two complementary issues relevant to storage conditions for medicinal products.

The first issue is scientific and climatic evolution since 2007, which requires more precise, stability-based storage statements.

The second issue is the lack of regulatory clarity and ambiguity in current terminology. The current EMA guideline CPMP/QWP/609/96/Rev 2, while grounded in ICH stability principles, predates the increasing focus on climate change, complex global distribution, and explicit risk-based approaches. In particular, the notion of “no special storage conditions” may be overly vague and easily misinterpreted by both patients and healthcare professionals.

The proposed revision CPMP/QWP/609/96/Rev 3 addresses these issues by:

- Reinforcing the requirement that storage statements be explicitly based on stability data and climatic realities;
- Eliminating vague temperature descriptors;
- Standardising core storage statements that are practical, harmonised, and numerically defined (e.g., 25ºC or 30ºC);
- Aligning with contemporary ICH and WHO expectations;
- Expanding guidance for sterile products, moisture- and light-sensitive products, and in-use stability;
- Introducing structured contingency instructions for temperature excursions, which is increasingly important under climate-related stress and power failures.

**Limitations:** The guideline proposal itself is a conceptual and regulatory exercise. Formal regulatory adoption would require broader stakeholder consultation, impact assessment, and possible alignment with additional EU and international initiatives.

## Conclusions

Revision of the EMA guideline on the declaration of storage conditions in product information for medicinal products and active substances is scientifically warranted and timely. The proposed update to CPMP/QWP/609/96 (Rev.2 to Rev.3):

- Aligns the scientific and regulatory rationale for storage statements with current ICH and WHO guidance;
- Increases the specificity, harmonization, and usability (user-friendly) of storage instructions;
- Explicitly incorporates considerations of current climatic variability, stability science, climate change, and supply-chain risk;
- Provides detailed standardized wording templates covering a broad range of product types, use cases, and contingency scenarios, to enhance patient and healthcare professional understanding.

Collectively, this proposal is intended to contribute constructively to future EMA deliberations and ultimately to improved support for more consistent storage labelling practices and ultimately to improved protection of medicinal product quality and patient safety.
